# An innovative surgical approach: suture fixation of the levonorgestrel-releasing intrauterine system in the treatment of adenomyosis

**DOI:** 10.1186/s12905-022-01932-6

**Published:** 2022-11-16

**Authors:** Huizhi Zhang, BenBen Cao, Jinyi Tong, Jialu Guo, Jianfeng Zheng, Linling Zhu, Zheng Niu, Li Chen

**Affiliations:** 1 Department of Gynecology, Chun’an County Maternity and Childcare Hospital, Hangzhou, China; 2 Department of Gynecology, Affiliated Hangzhou First People’s Hospital, Hangzhou, China; 3 Department of Gynecology, Affiliated Hangzhou Maternity and Childcare Hospital, Hangzhou First People’s Hospital, No. 261 Huansha Road, Hangzhou, 310003 Zhejiang Province China; 4 Department of Gynecology, The First Affiliated Hospital of Zhejiang University School of Medicine, Hangzhou, China

**Keywords:** Levonorgestrel-releasing intrauterine system, Adenomyosis, Hysteroscopic cold knife surgery system

## Abstract

**Background:**

Placement of a levonorgestrel-releasing intrauterine system (LNG-IUS) is an effective treatment for adenomyosis, especially for patients who have severe dysmenorrhea symptoms but a strong desire to preserve fertility. Nonetheless, for patients with adenomyosis accompanied by an enlarged uterus, expulsion of the ring is a troublesome problem. In this study, we sewed and fixed the LNG-IUS in the uterus, which provides a good solution to this problem.

**Methods:**

In this prospective case series approved by the Ethics Committee of Hangzhou Women’s Hospital, 12 patients with adenomyosis were successfully enrolled after providing informed consent, and all patients underwent long-term postoperative follow-up.

**Results:**

Twelve patients with adenomyosis underwent suture fixation with an LNG-IUS, and during the long-term postoperative follow-up, every patient experienced complete remission of their symptoms: a significant decrease in menstrual flow, relief of dysmenorrhea, and improvement in quality of life. Only one person reported expulsion a year later.

**Conclusion:**

In patients with adenomyosis suffering from dysmenorrhea or excessive menstrual blood loss, suture fixation of an LNG-IUS using the hysteroscopic cold knife surgery system is a minimally invasive and effective alternative treatment for adenomyosis and decreases the risk of LNG-IUS expulsion.

## Background

Adenomyosis is a heterogeneous gynecologic condition with a range of clinical symptoms, the most common being heavy menstrual bleeding and dysmenorrhea. At present, total hysterectomy is considered the most effective treatment for uterine adenomyosis [1]. However, given that many patients have a strong desire to preserve the uterus, the treatment of adenomyosis should be personalized according to a woman's age, reproductive status, and clinical symptoms. Currently, medical therapy shows increasing efficacy in patients requiring control of symptoms or fertility treatments [2]. Among these, the LNG-IUS is the most promising medical therapy, according to the literature, due to its ability to suppress hormones to improve symptoms with a low profile of adverse effects while enabling women to maintain fertility [3]. However, there are some limitations to the suitability of the LNG-IUS for women with adenomyosis, as adenomyosis causes distortion of the uterine cavity or an enlarged uterine cavity. Although the uterine size is not considered one of the contraindications for the use of intrauterine devices (IUDs, including the LNG-IUS), it is recommended that all IUDs be inserted in uteri at a depth below 9 cm [4]. As Park et al. reported, 37.5% of patients with large symptomatic adenomyosis experienced LNG-IUS expulsion, and almost all of which occurred during the first 6 months [5].

To address this problem, in our study, we proactively performed suture fixation of the LNG-IUS in the uterine cavity by using the hysteroscopic cold knife surgery system (HCSS).

## Methods

### Patients

The study was approved by the Ethics Committee of Hangzhou Women’s Hospital and started in January 2021. Twelve patients with adenomyosis have providing informed consent and been successfully enrolled thus far.

- Inclusion criteria: (a) had menorrhagia or dysmenorrhea and met the diagnosis of "adenomyosis"; (b) had a strong desire to preserve the uterus; and c) had no fertility requirements in the short term.
- Preferred criteria: (a) enlarged uterus; (b) history of expulsion.
- Exclusion criteria: (a) contraindications to use of the LNG-IUS; (b) contraindications to hysteroscopic surgery; (c) other diseases of the reproductive tract (malignant tumors, deformities, or acute infections); (d) severe heart, lung, liver, and kidney insufficiency; (e) had received gonadotropin-releasing hormone a (GnRG-a) treatment within the previous 6 months; and (f) reluctance to participate in this study.

### Materials

The materials used in this study included the following: the levonorgestrel-releasing intrauterine system (Mirena Manufacturer: Bayer; Standard Chinese Medicine: J20090144. 52 mg; 20 µg/24 h), hysteroscopic cold knife surgery system (Hangzhou Sode Medical Equipment Co., Ltd.), endoscopic scissors, endoscopic needle holder, and the STRA TAFIXTM Spiral PGA-PCL (SXMD1B405, Shanghai, Johnson & Johnson).

### Operative procedure

**Fig. 1:**
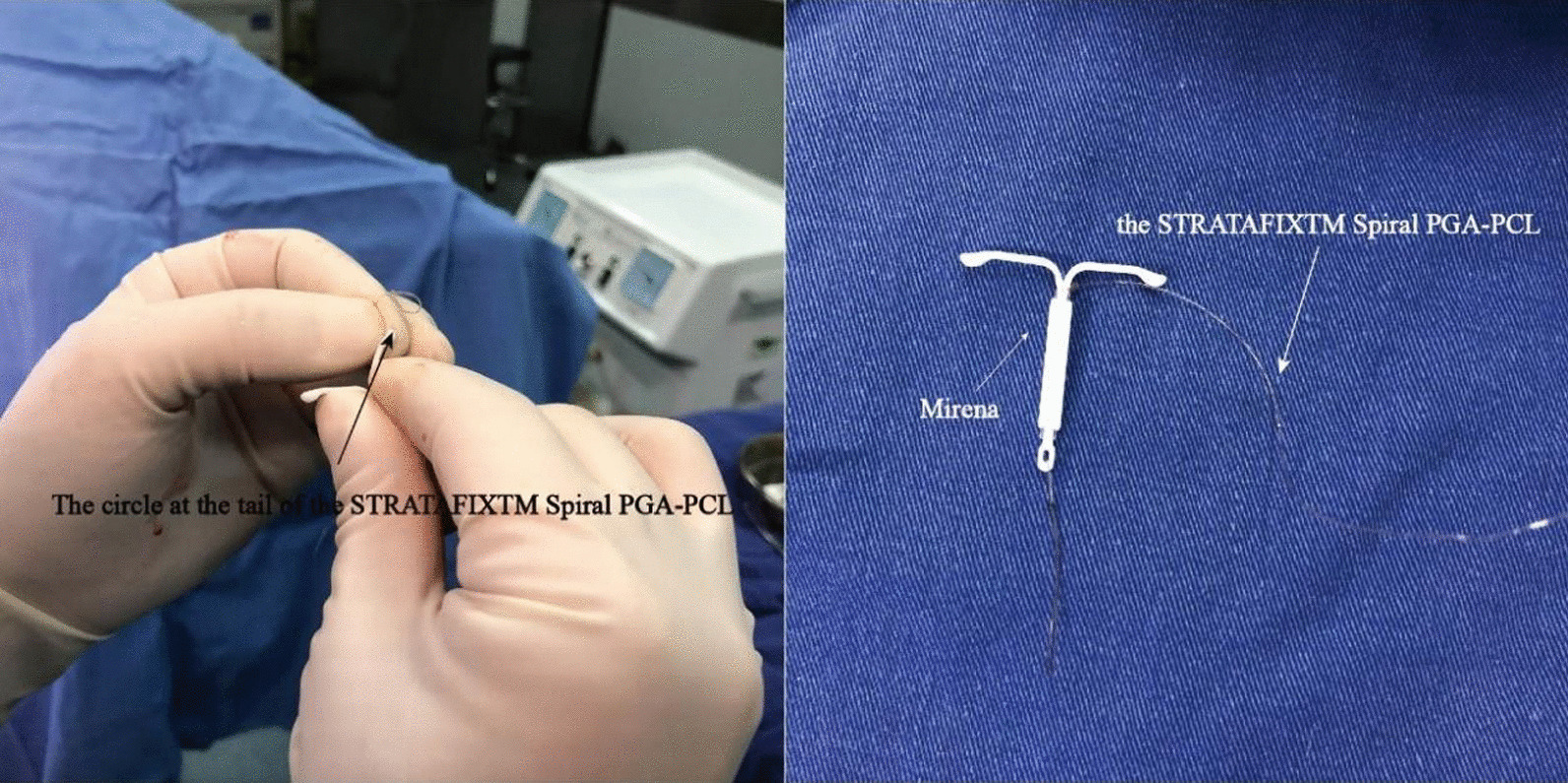
The intersection of the arms of the Mirena device fixed in a circle at the tail of the STRATAFIXTM Spiral PGA-PCL

Under general anesthesia with the patient lying in the lithotomy position, hysteroscopy was performed using the HCSS (Hangzhou Sode Medical Equipment Co., Ltd.). The HCSS is equipped with a diameter of 4 mm working channel, hysteroscopic surgical instruments such as scissors and grasping forceps can be inserted through it. We first assessed the uterine cavity morphology and endometrial condition. If polyps, endometrial thickening, intrauterine adhesions, etc., were found, corresponding treatments were performed first. If no abnormality was found, the appropriate amount of endometrial tissue was scraped off to create a good suture fixation environment that was clearly visible. We then fixed the intersection of the arms of the LNG-IUS in the circle at the tail of the STRA TAFIXTM Spiral PGA-PCL (Fig. 1) and sutured it on the posterior wall of the uterus with a laparoscopic gripper. Finally, we snipped the Spiral PGA-PCL at the external uterine orifice.

### Measurements

- Pictorial Blood Loss Assessment Chart (PBAC)
  Blood loss was assessed according to the degree of blood staining on each sanitary napkin as follows: Mild: blood-stained area ≤ 1/3 of the entire sanitary napkin area; Moderate: blood-stained area occupies 1/3–3/5 of the entire sanitary napkin area; Severe: the blood-stained area is basically the entire sanitary napkin. The corresponding scores were 1, 5, and 20 points, respectively. In addition, blood clots smaller than a coin were given 1 point, while larger ones were given 5 points. The total score was calculated based on the score of each sanitary napkin and the numbers. A total score of 100 or more was considered to be menorrhagia.
- Visual Analog Scale (VAS)
  The degree of dysmenorrhea was assessed as follows: no pain (0 points), mild pain (1–3 points), moderate pain (4–6 points), and severe pain (7–10 points).
- Nottingham Health Profile (NHP)

The NHP scale was used to evaluate the quality of life of patients and has good reliability. It provides a brief indication of a patient's perceived emotional, social, and physical health [6]. It is filled out by the patient before and after treatment. The scores on the NHP domains range from 0 (best health state) to 100 (worst health state).

### Postoperative follow-up

The status of the LNG-IUS was evaluated by TVS. Menstrual flow was quantified with a pictorial blood assessment chart (PBAC), and the degree of dysmenorrhea was evaluated on a 100 mm visual analog scale (VAS). Both the PBAC scores and the VAS scores were recorded before and 1, 3, and 6 months after insertion of the LNG-IUS. Adverse reactions to the LNG-IUD were recorded in the medical records, including menstrual pattern changes, abdominal discomfort, abnormal vaginal discharge, and climacteric symptoms.

## Results

In this study, twelve patients received LNG-IUS suture fixation, and all had symptoms of dysmenorrhea (4/12), excessive menstrual flow (3/12), or both (5/12). The depth of their uterine cavity was greater than 9 cm, with an average of 9.79 cm. Three patients had a history of expulsion, with an average time of expulsion of approximately one month after placement.

After suture fixation of the levonorgestrel-releasing intrauterine system, when patients come for follow-up, we ask them about remission of symptoms and monitor the position of the LNG-IUS through ultrasound. The following three pictures vividly demonstrate the changes in menstrual flow, dysmenorrhea, and quality of life after surgery.

**Fig. 2:**
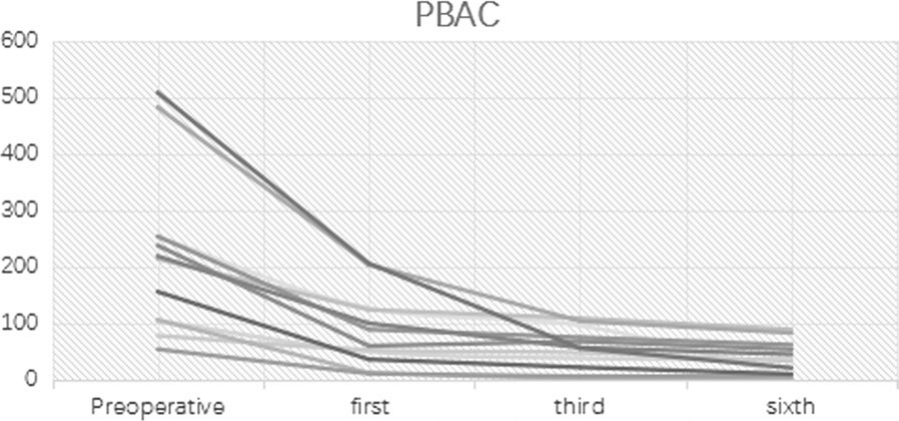
PBAC score changes of the 12 patients

**Fig. 3:**
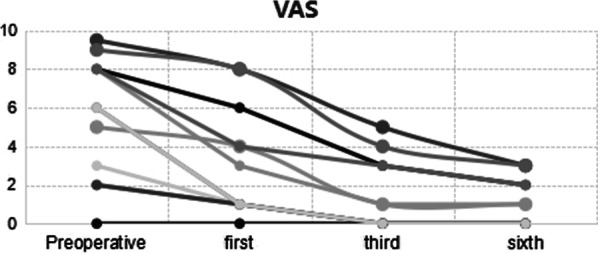
VAS score changes of the 12 patients

**Fig. 4:**
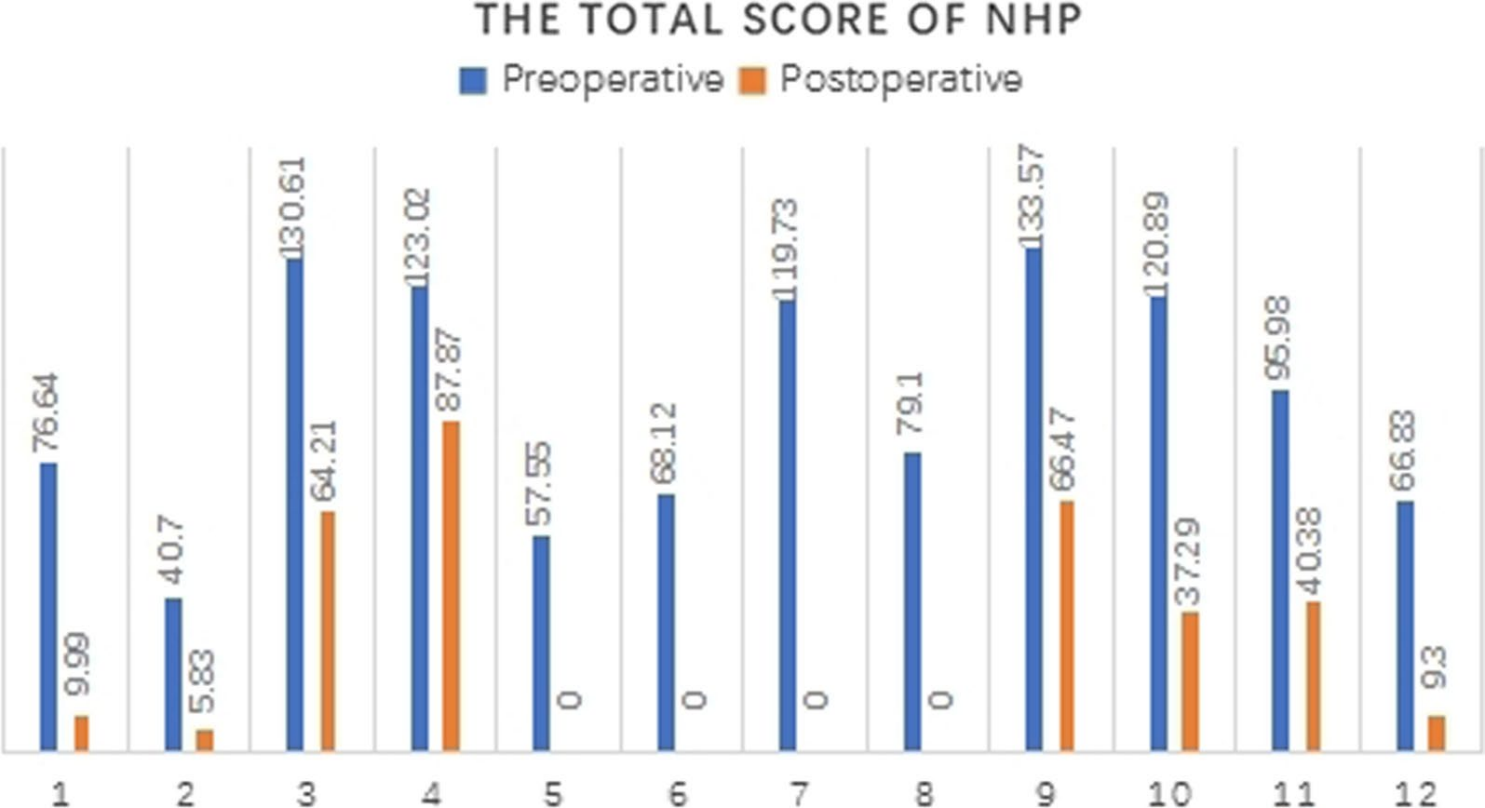
Total NHP score

As seen, the PBAC scores (Fig. 2) and the VAS scores (Fig. 3) began to decrease in the first month after LNG-IUS fixation, according to the downward trend of the broken line in the figure, it can be clearly seen that in the six months from the operation to follow-up, the menstrual volume was significantly reduced, and dysmenorrhea was significantly relieved. The NHP scores (Fig. 4) before and after surgery, indicating that the quality of life of the 12 patients greatly improved after surgery. Among them, four patients (33.3%) had a postoperative NHP score of zero, indicating that they were completely relieved from the disease, and their lives were no different from those of normal people. Besides, four patients complained of prolonged vaginal spotting, one of whom experienced hot flashes. Three women reported abnormal vaginal discharge. Regardless, no patient requested premature removal of the LNG-IUS.

Unfortunately, during follow-up, a patient experienced expulsion of the LNG-IUS after twelve months. Two other patients also exhibited a downward shift in the position of the LNG-IUS in the uterus on ultrasound; one occurred in the fourth month after the operation, and the other occurred in the sixteenth month. After identifying the problem, we thoroughly analyzed it and developed an improvement plan, which will be explained in detail in the follow-up discussion.

## Discussion

Adenomyosis is a common gynecological disorder characterized by invasion of endometrial glands and stroma into the myometrium, which results clinically in abnormal uterine bleeding, pelvic pain, and infertility [7]. Long-term chronic blood loss can even lead to anemia, as well as psychological disorders due to chronic menstrual pain. As the disease has a negative impact on quality of life in terms of menstrual symptoms, fertility, and pregnancy outcome, a long-term and effective treatments for adenomyosis is necessary [8]. Moreover, both adenomyosis and endometrial cancer (EC) can cause abnormal uterine bleeding (AUB) according to the FIGO classification system (PALM-COEIN) [9]. Some people have noted that the histological features of adenomyosis are somewhat similar to those of infiltrating endometrial cancer, and even diagnostic hysteroscopy has certain disadvantages [10]. Some scholars have even proposed that adenomyosis may be associated with an increased risk of developing EC since they share several altered molecular pathways and are both associated with similar local microenvironments [11]. Therefore, there is an urgent need for long-term and effective treatments for adenomyosis. Relevant studies have shown that the levonorgestrel-releasing intrauterine system can effectively treat the symptoms of abnormal uterine bleeding and dysmenorrhea in the long-term management of adenomyosis [5, 12, 13]. This is the theoretical basis for our research: the LNG-IUS is indeed a practical option for women requiring fertility-sparing management of adenomyosis.

However, high rates of LNG-IUS expulsion may be a deterrent to patients who have discontinued treatment of adenomyosis, especially those with a large uterus volume (> 150 mL) [14]. A prospective longitudinal study over a 60-month follow-up period (the longest follow-up of LNG-IUS for the treatment of adenomyosis to date) reported that the expulsion rate reached up to 21.8% when the patient had undergone pretreatment with GnRHa; approximately one-third of the patients had the LNG-IUS replaced, and more than half of them chose another therapy [13], which is a waste of health care resources and increases anxiety among patients.

Some studies have confirmed that pretreatment with gonadotropin-releasing hormone analog (GnRHa) is beneficial to a certain extent in reducing the size of the uterus [13, 15, 16], and it does greatly reduce the incidence of LNG-IUS expulsion. On the other hand, the adverse effects of GnRHa (e.g., menopausal symptoms and risk of osteoporosis) and the cost–benefit profile should be addressed as appropriate. In contrast, our approach is more advantageous due to fewer side effects and a lower cost. We also observed some side effects during our follow-up, but they had already been observed in the process of using the LNG-IUS without fixation [13] and were considered acceptable and tolerable.

To date, few studies have reported the role of LNG-IUS fixation. Currently on the market, there is Gynefix, which is specially designed to prevent the expulsion of IUDs, but Gynefix itself does not have a drug treatment effect comparable to that of Mirena and cannot replace Mirena at this point. It was asked whether the Mirena could be designed in the style of Gynefix to better address this problem. Practically, the idea is feasible, but due to the patent restrictions of the Mirena R&D company, the advancement of this design is nowhere in sight. Therefore, in our study, we proactively performed suture fixation of the LNG-IUS in the uterine cavity by using the hysteroscopic cold knife surgery system (HCSS), and satisfactory results were obtained. The symptom relief of patients with adenomyosis is similar to that in most prospective and retrospective studies. Notably, the incidence of expulsion of the LNG-IUS during our follow-up observation period was extremely low (1/12).

We stitched the LNG-IUS on the posterior wall of the uterus and fixed it; theoretically, it was firmly fixed and could not be expelled. However, as stated in our results, one patient expelled the LNG-IUS after twelve months of follow-up, and two other patients showed a downward shift of the LNG-IUS in the uterus. The suture material used in this trial was polydioxone (PDO), which usually takes approximately 180–240 days to be absorbed [17]. Given this characteristic, we have reason to believe that the expulsion of the LNG-IUS was due to the absorption of the suture.

**Fig. 5:**
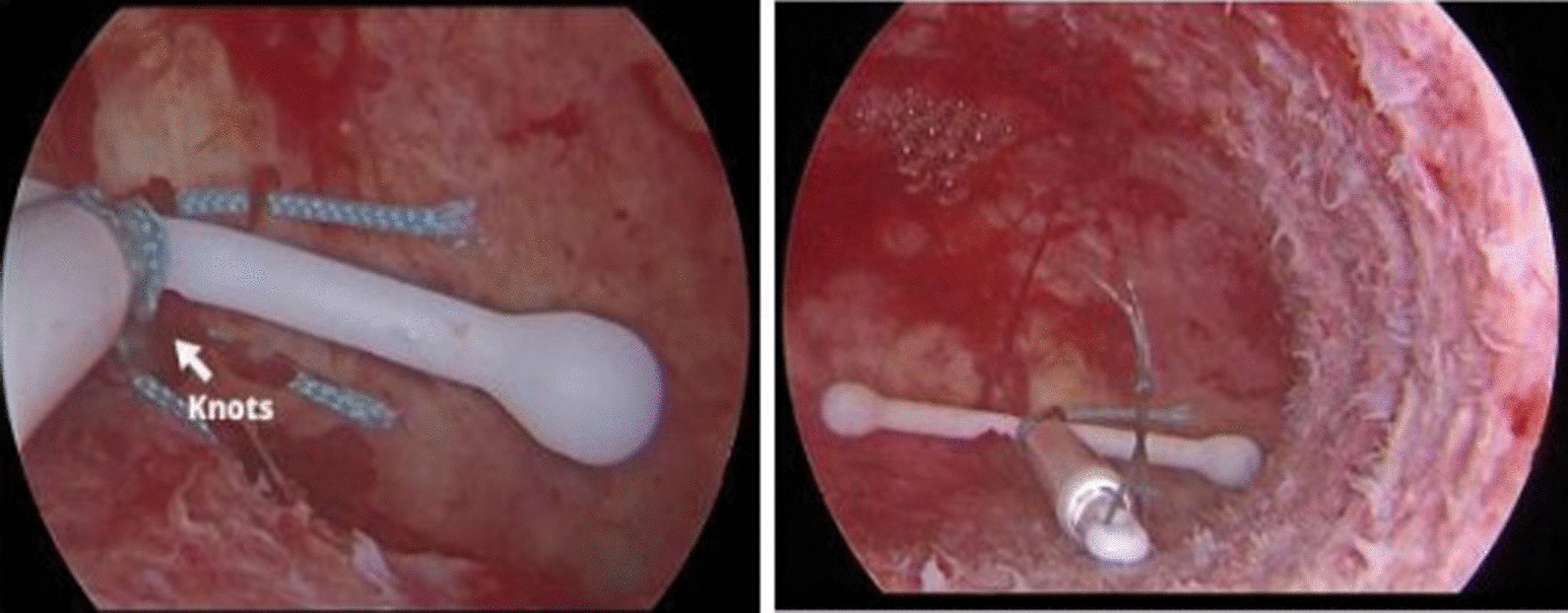
The ING-IUS was fixed in the uterus

Therefore, we made some adjustments involving replacement of the original absorbable thread with a nonabsorbable surgical suture (ETHICON, INC 2006) and improved the method of fixation. First, the nonabsorbable suture was tied to the arms and stem of the LNG-IUS. After the suture was fixed on the wall of the uterus, the suture was pulled to send the LNG-IUS into the uterus, and the knot was tied outside. Finally, the knot pushing device was used to push and fix the knot tightly (Fig. 5). This improvement made the procedure a great success, and our team has successfully published the operation video so that gynecologists can learn and discuss the technique [18].

The improved fixation of the LNG-IUS not only solves the previous problem of expulsion due to suture absorption but also achieves an ideal expulsion rate of zero. To date, we have completed more than 70 cases of suture fixation of the LNG-IUS with this improved approach. The patient group is being followed up, with the longest follow-up time being 10 months, and no expulsion has occurred. This improved approach simplifies the process of suturing and knotting in the uterus and involves a better method of knotting in vitro. On the one hand, the operation time is greatly shortened; on the other hand, the method involving knotting outside the body avoids repeatedly entering and leaving the uterus and reduces the risk of intrauterine infection. In addition, this improved approach greatly reduces the difficulty of the operation, and it is easy for clinicians to get started. Currently, our surgical team completes a case of suture fixation of the LNG-IUS in an average of 10 to 15 min, which is equivalent to completing a simple endometrial polypectomy. The operation cost is equivalent to that of an ordinary hysteroscopic surgery and placement of an IUD, and there is no additional operation cost. More generally, suture fixation of the LNG-IUS is the only surgical technique that truly achieves fixation thus far at home or abroad. With the improvements made by our team, the operation is simplified and easy to implement widely, and the cost is reasonable. Compared with patients who experience multiple expulsions and then require replacement of the Mirena multiple times, patients treated with our method benefit from a simple hysteroscopic operation for at least 5 years (the validity period of Mirena), which not only saves the economic cost of multiple purchases of Mirena devices but also alleviates patient worry about the incidence of expulsion, so that the vast number of patients with adenomyosis are no longer in deep pain.

Our project is a case series about a new technique and has several limitations. First, this was a noncomparative prospective study. The number of cases included is relatively small, and the number of evaluation indicators could be further increased to include metrics such as changes in hemoglobin, CA125, uterine size, and so on. However, satisfactory results have been achieved in stages, and patients who have experienced expulsion have also received LNG-IUS fixation with the new suture fixation. We will continue to improve this innovative surgical method. A more perfect and rigorous RCT (Hangzhou Medical and Health Technology Project: Z20210010) is underway to evaluate the efficacy of LNG-IUS fixation, aiming to solve the problem of LNG-IUD expulsion.

## Conclusion

Suture fixation of the levonorgestrel-releasing intrauterine system better addresses the drawbacks of expulsion through a minimally invasive operation. At the same time, similar to normal placement of an LNG-IUS, it effectively relieves the dysmenorrhea and heavy bleeding symptoms of adenomyosis.

## Data Availability

The datasets used and/or analysed during the current study are available from the corresponding author on reasonable request.

## Abbreviations

LNG-IUS: Levonorgestrel-releasing intrauterine system
HCSS: Hysteroscopic cold knife surgery system
PBAC: Pictorial Blood Loss Assessment Chart
VAS: Visual Analog Scale
NHP: Nottingham Health Profile
EC: Endometrial cancer
AUB: Abnormal uterine bleeding
PALM-COEIN: The FIGO classification system
GnRHa: Gonadotropin-releasing hormone analog
